# Commentary on a GWAS: HDAC9 and the risk for ischaemic stroke

**DOI:** 10.1186/1741-7015-10-70

**Published:** 2012-07-09

**Authors:** Werner Hacke, Caspar Grond-Ginsbach

**Affiliations:** 1 Department of Neurology, University of Heidelberg, D-69120 Heidelberg, Germany; 2 Department of Neurology, University of Heidelberg, D-69120 Heidelberg, Germany

## Abstract

Modifiable risk factors like obesity, hypertension, smoking, physical inactivity or atrial fibrillation account for a significant proportion of the risk for ischaemic stroke, but genetic variation is also believed to contribute to the risk, although few genetic risk variants were identified to date. Common clinical subtypes of stroke are caused by cardiac embolism, large artery atherosclerosis and small cerebral vessel disease. Each of these underlying pathologies may have a specific genetic architecture.

Previous genome-wide association studies (GWAS) showed association of variants near *PITX2 *and *ZFHX3 *with atrial fibrillation and stroke. *ANRIL *(antisense Non-coding RNA in the *INK4 *Locus (harboring the *CDKN2A/B *genes)) variants were related to a variety of vascular diseases (myocardial infarction, aortic and intracranial aneurysm), including ischaemic stroke. Now a recent GWAS published in *Nature Genetics *confirmed these previous associations, analyzed the specificity of the previous associations with particular stroke subtypes and identified a new association between *HDAC9 *and large vessel stroke. The findings suggest that well-recognized clinical stroke subtypes correspond to distinct aetiological entities. However, the molecular pathways that are affected by the identified genetic variants are not yet pinpointed, and the observed associations apply only for some, but not all victims of a specific stroke aetiology.

## Background

The International Stroke Genetics Consortium and the Wellcome Trust Case Control Consortium 2 published the largest genome-wide association study (GWAS) for ischaemic stroke carried out to date [1]. Ischaemic stroke is the second leading cause of death and the major cause of long-term disability worldwide [2]. The risk for stroke increases with age and with risk factors like cigarette smoking, obesity, physical inactivity, hypertension, atherosclerosis, atrial fibrillation, a family history of stroke or a previous stroke or TIA [3]. However, not all brain infarcts are alike. Rather, they have multiple, sometimes competing, underlying aetiologies and subtypes. Five stroke subtypes were defined according to the TOAST (Trial of Org 10172 in Acute Stroke Treatment) classification [4], using clinical and diagnostic features: 1) large-artery atherosclerosis (large vessel disease, LVD), 2) cardioembolism (CE), 3) small-vessel occlusion (small vessel disease, SVD), 4) stroke of other determined etiology (rare underlying causes such as arterial dissection, inherited diseases of the coagulation system or vasculitis), and 5) stroke of undetermined etiology. Patients with LVD, SVD and CE stroke subtypes have distinct symptoms, distinct risk profiles and subtype-specific risks for recurrent events or events in first degree relatives [5,6]. Stroke risk modification and stroke prevention remain major items in health care. Primary and secondary stroke prevention strategies differ according to stroke subtype, for example, anticoagulation for cardioembolic strokes, platelet inhibition for large vessel disease, antihypertensive treatment and statins for most subtypes [6].

In some patients, despite extensive search, no underlying causes are found and increasingly, we identify overlapping etiologies, such as the combination of large vessel (carotid) arteriosclerosis and atrial fibrillation as a potential source of a cardioembolism. Even the subgroup LVD-stroke is heterogeneous and far from being a simple homogeneous phenotype. Many readers might feel that LVD mainly refers to carotid bifurcation occlusive disease. However, thrombogenic aortic disease, vertebrobasilar occlusive disease (which appears to be different from carotid disease), distal carotid stenosis and intracranial occlusive disease (more frequent among Asians and Afro-Americans) are all part of the LVD-definition.

## Genetic association studies of ischaemic stroke

The identification of genetic variants that modify the risk for stroke is still in its infancy. Inherited stroke syndromes with identified disease-causing mutations exist, but are rare [7]. The majority of strokes are thought to be associated with a complex variety of interacting risk factors, related to co-morbidities, life style, baseline characteristics and genetic variants [8]. The impact of environmental and modifiable risk factors on stroke is well recognized, albeit difficult to capture in its whole complexity. On the contrary, modern genetic technologies like high density single nucleotide polymorphism (SNP) microarrays or second generation sequencing approaches permit the precise, rapid and standardized documentation of a growing number of individual genetic variants - at falling prices.

A GWAS is an observational study in which genetic variation is compared between people with a particular condition (cases) and unaffected control individuals. Because genetic variants are assigned at random from parents to offspring (Mendel's law of segregation) before birth (prospectively), a genetic case control study can be thought of as a "natural", randomized controlled trial.

The GWAS technology is based on the popular hypothesis that common diseases are associated with common genetic variants [9]. However, GWAS results probably explain only a few percent of the apparent genetic variance contributing to common diseases [10]. Some limitations of the GWAS approach (lack of power due to insufficient sample size, phenotypic heterogeneity) may be solved, but others (only selected single nucleotide variants with frequent alleles are genotyped) are intrinsic to the microarray technology. It is to be expected that other genetic techniques (whole exome sequencing studies or analysis of rare copy number variation [11]) will identify other types of genetic variation (*de novo *mutations, rare mutations, structural aberrations) that contribute to the risk for stroke [12,13].

The currently published GWAS is a milestone in stroke genetics for several reasons: it demonstrates the power of very large multicenter study samples, advocates the study of distinct stroke subtypes, replicates findings of previous stroke GWAS and identifies a new associated genetic variant. Moreover, the authors introduce a statistical model to demonstrate the subtype-specificity of the identified associations. Accordingly, the association near the *PITX2 *and *ZNFX3 *genes was confirmed to be specific for cardioembolic stroke, as to be expected [14], since these variants were associated with atrial fibrillation, a major risk factor for cardiac embolism. The association of the *ANRIL *locus (chromosome 9p21 locus) with LVD seemed less specific. Indeed, alleles of this locus were associated with a broad variety of vascular conditions, including myocardial infarction, type 2 diabetes mellitus, aortic aneurysm and intracranial aneurysm [15,16].

SNP microarray studies start from the "common diseases/common genetic variants" hypothesis. However, the current GWAS study suggests that stroke genetics develops towards the study of multiple distinct stroke subtypes instead of a "common stroke" phenotype. Future analysis of well defined stroke subtypes and of a combination of multiple risk alleles [17] may lead to the identification of high-risk multilocus genotype patterns, but the population frequency of such allelic combinations will be low. Hence, fragmentation of the stroke phenotype into genetically homogeneous subtypes and their association with specific genetic risk profiles may lead away from the GWAS methodology and from the "common disease/common genetic variants" hypothesis towards the identification of personalized genetic blueprints as predictors of individual disease risks.

## Conclusion

The currently published GWAS of ischaemic stroke advocated the study of distinct stroke subtypes, replicated findings of previous studies and identified a new association between the A-allele of rs11984041 in *HDAC9 *and ischaemic stroke caused by LVD. The *HDAC9 *gene encodes histone deacetylase 9, which is known to be involved in heart and muscle tissue development. Carriers of the A-allele (about 9% of the study population) have a 1.4 times increased risk for stroke caused by LVD, and homozygous carriers (1% of the population) have a two-fold risk. The underlying molecular pathways explaining the association between *HDAC9 *and large vessel stroke are currently unknown.

Further studies of common genetic variants in large samples of patients with stroke subtypes or intermediate phenotpyes and future analysis of rare genetic variants may identify molecular pathways involved in stroke risk. Recent GWAS findings, however, do not yet have direct implications for stroke risk stratification [18,19].

## Abbreviations

*ANRIL*: Antisense Non-coding RNA in the *INK4 *Locus (harboring the *CDKN2A/B *genes); CE: cardio-embolism; GWAS: genome wide association study; *HDAC9*: gene encoding histone deacetylase 9; LVD: large vessel disease; *PITX2*: gene encoding paired-like homeodomain 2/pituitary homeobox2; SNP: single nucleotide polymorphism; SVD: small vessel disease; TIA: transient ischaemic attack; TOAST classification: classification of stroke subtypes according to the Trial of Org 10172 in Acute Stroke Treatment; *ZFHX3*: gene encoding zinc finger homeobox 3.

## Competing interests

The authors declare that they have no competing interests.

## Authors' contributions

WH and CGG contributed equally to this commentary. Both authors were involved in the development and writing of this manuscript and approved the final version of the manuscript.

## Authors' information

Werner Hacke is Medical Director of the Neurology Clinic at Heidelberg University Hospital. Caspar Grond-Ginsbach is Head of the Laboratory of Molecular Genetics of Stroke at the Department of Neurology of the Heidelberg University Hospital.

## Pre-publication history

The pre-publication history for this paper can be accessed here:

http://www.biomedcentral.com/1741-7015/10/70/prepub
